# Supplementary material for: Commensal lifestyle regulated by a negative feedback loop between Arabidopsis ROS and the bacterial T2SS
Source: Nat Commun. 2024 Jan 11;15:456. doi: 10.1038/s41467-024-44724-2 (PMC10784570; doi:10.1038/s41467-024-44724-2)
Supplement: Supplementary file 1 — Supplementary Information [file 41467_2024_44724_MOESM1_ESM.pdf]

Commensal lifestyle regulated by a negative feedback loop between *Arabidopsis* ROS and the bacterial T2SS

Frederickson Entila[1,2], Xiaowei Han[1,3,4], Akira Mine[5,6], Paul Schulze-Lefert[2], Kenichi Tsuda[1,2,3,4*]

[1]National Key Laboratory of Agricultural Microbiology, Hubei Hongshan Laboratory, Hubei Key Laboratory of Plant Pathology, College of Plant Science and Technology, Huazhong Agricultural University, Wuhan 430070, China.

[2]Department of Plant Microbe Interactions, Max Planck Institute for Plant Breeding Research, Carl-von-Linne-Weg 10, Cologne 50829, Germany

[3]Shenzhen Institute of Nutrition and Health, Huazhong Agricultural University, Wuhan 430070, China.

[4]Shenzhen Branch, Guangdong Laboratory of Lingnan Modern Agriculture, Genome Analysis Laboratory of the Ministry of Agriculture and Rural Affairs, Agricultural Genomics Institute at Shenzhen, Chinese Academy of Agricultural Sciences, Shenzhen, Guangdong 518120, China

[5]JST PRESTO, Kawaguchi-shi, Saitama 332-0012, Japan

[6]Laboratory of Plant Pathology, Graduate School of Agriculture, Kyoto University, Kyoto 606-8502, Japan

[*]To whom correspondence may be addressed. Email: tsuda@mail.hzau.edu.cn

Supplementary Figures and legends

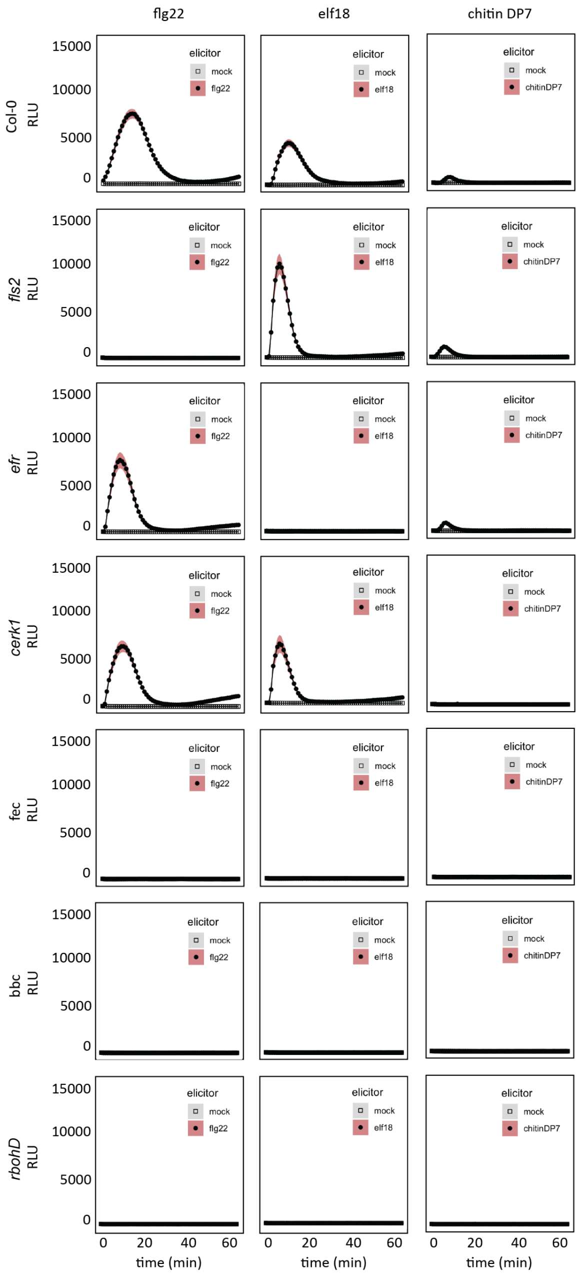

Supplementary Figure S1. ROS burst profile of immune-compromised mutants and Col-0 wildtype plants with MAMPs. Leaf discs from 5-to-6-week-old plants were treated with 1 µM of MAMPs, flg22, elf18, and chitinDP7. The immune-compromised mutant *fls2* lacks the receptor recognizing flg22, *efr* lacks the receptor for elf18, and *cerk1* lacks the co-receptor for chitinDP7; *fec* (*fls2 efr, cerk1*) and *bbc* (*bak1 bkk1 cerk1*) are triple mutants lacking the MAMP (co) receptor. Data from at least 2 independent experiments each with 8 biological replicates were used (for flg22, n=24 for Col-0 and 32 for the rest of the mutants; for elf18, n=24 for Col-0, *fec*, *bbc*, and *rbohD* while 32 for the rest; for chitin DP7, n=24 for all of the genotypes; for mock, n=216 for Col-0; 256 for *fls2*, *efr*, and *cerk1*; 176 for *fec* and *bbc*; and 72 for *rbohD*). Results are shown as line graphs using Locally Estimated Scatter Plot Smoothing (LOESS) with error bars and shadows indicating the standard errors of the mean.

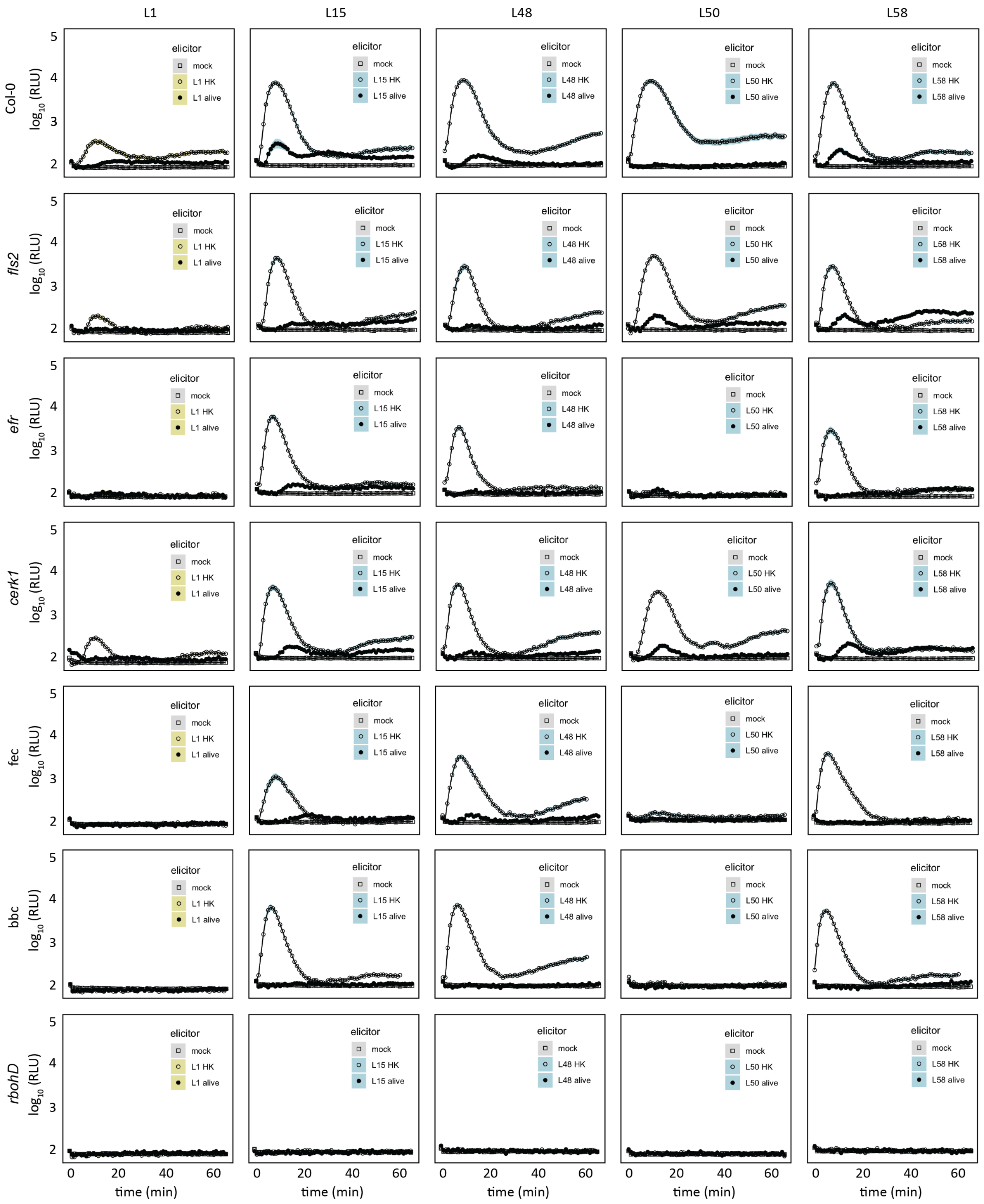

L1
L15
L48
L50
L58
Col-0
fls2
efr
cerk1
fec
bbc
rbohD
log10 (RLU)
5
4
3
2
elicitor
mock
L1 HK
L1 alive
L15 HK
L15 alive
L48 HK
L48 alive
L50 HK
L50 alive
L58 HK
L58 alive
0
20
40
60
time (min)

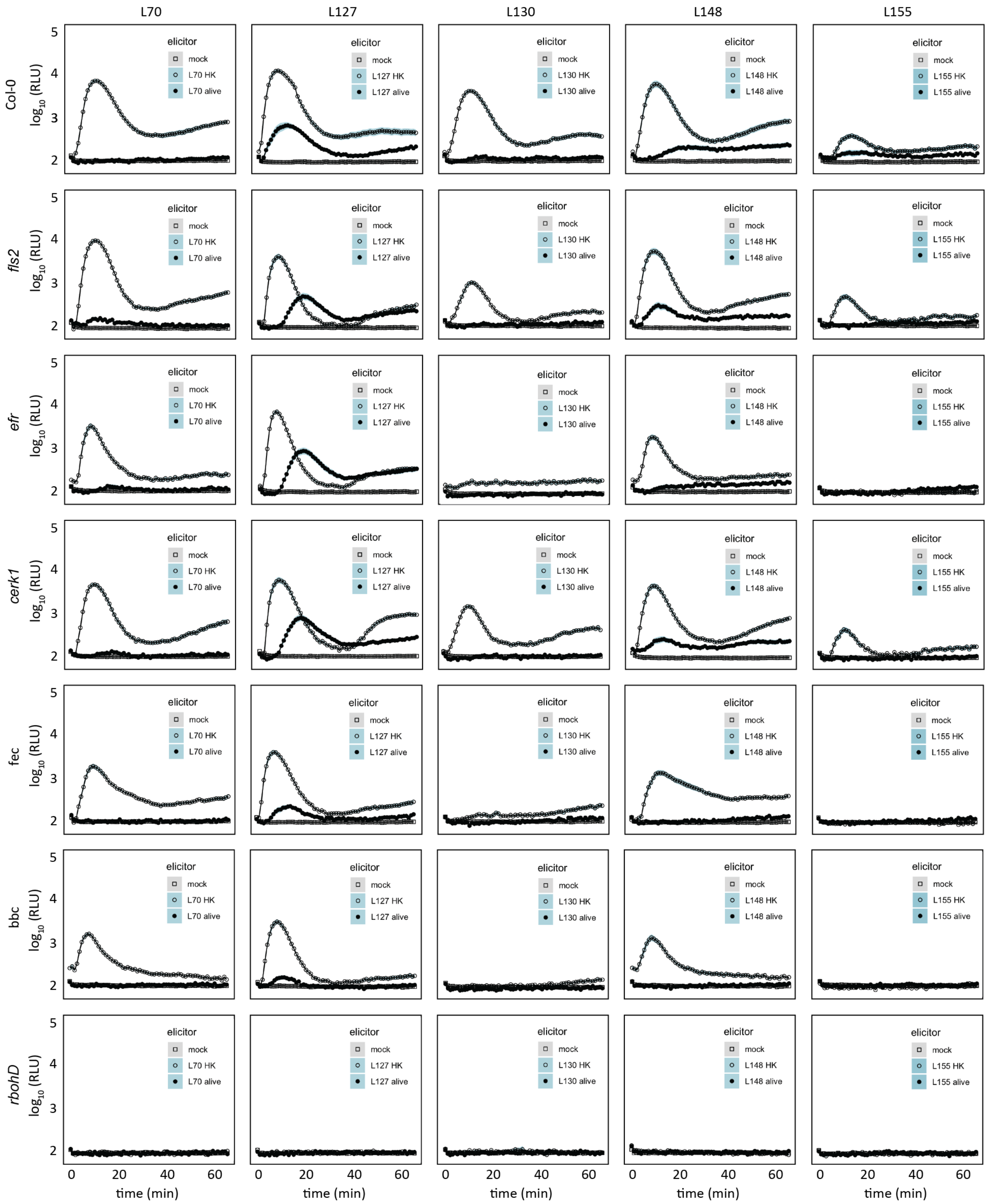
L70
L127
L130
L148
L155
Col-0
fls2
efr
cerk1
fec
bbc
rbohD
log10 (RLU)
5
4
3
2
elicitor
mock
L70 HK
L70 alive
L127 HK
L127 alive
L130 HK
L130 alive
L148 HK
L148 alive
L155 HK
L155 alive
0
20
40
60
time (min)

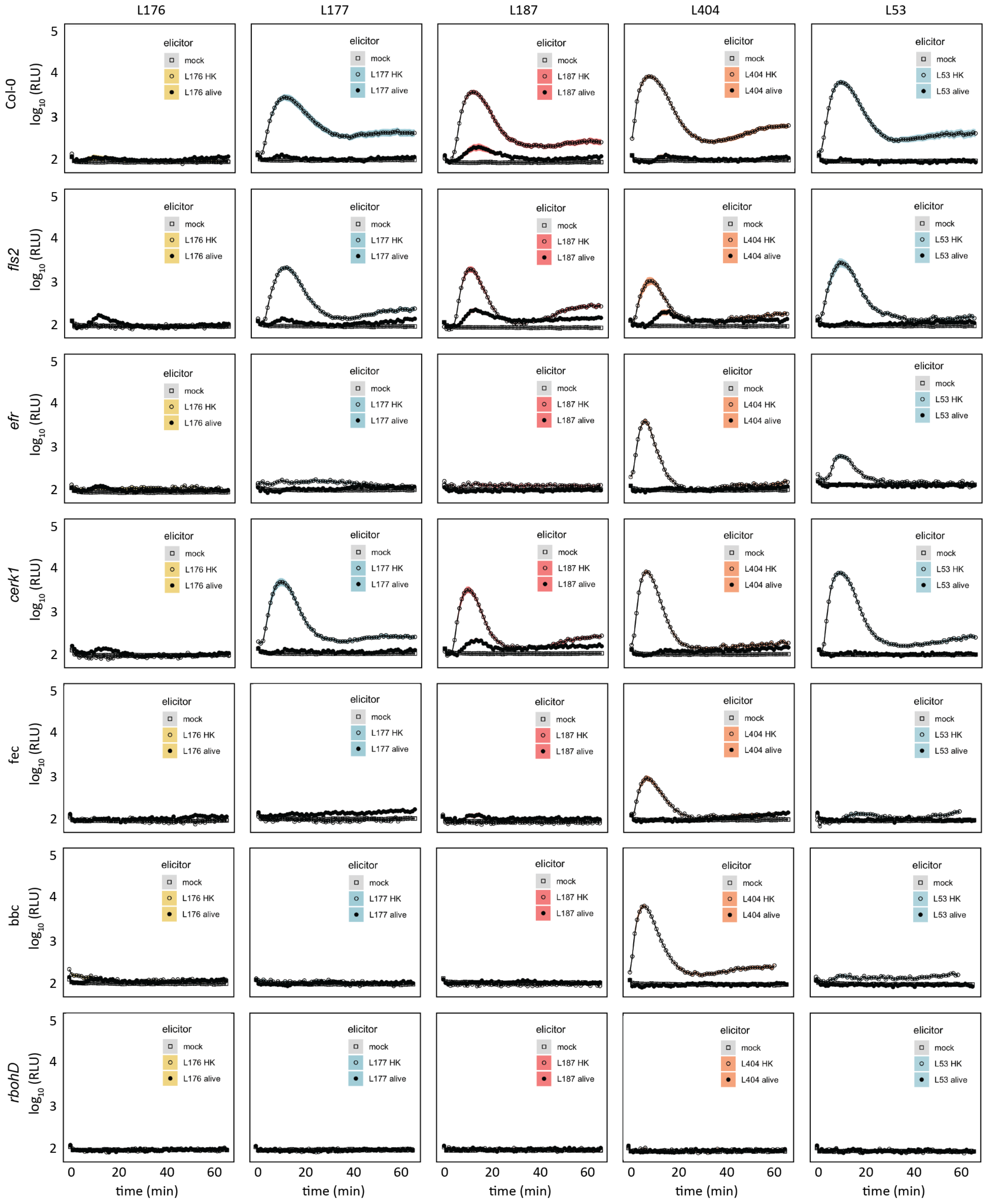

L176
L177
L187
L404
L53
Col-0
fls2
efr
cerk1
fec
bbc
rbohD
log10 (RLU)
5
4
3
2
elicitor
mock
L176 HK
L176 alive
L177 HK
L177 alive
L187 HK
L187 alive
L404 HK
L404 alive
L53 HK
L53 alive
0
20
40
60
time (min)

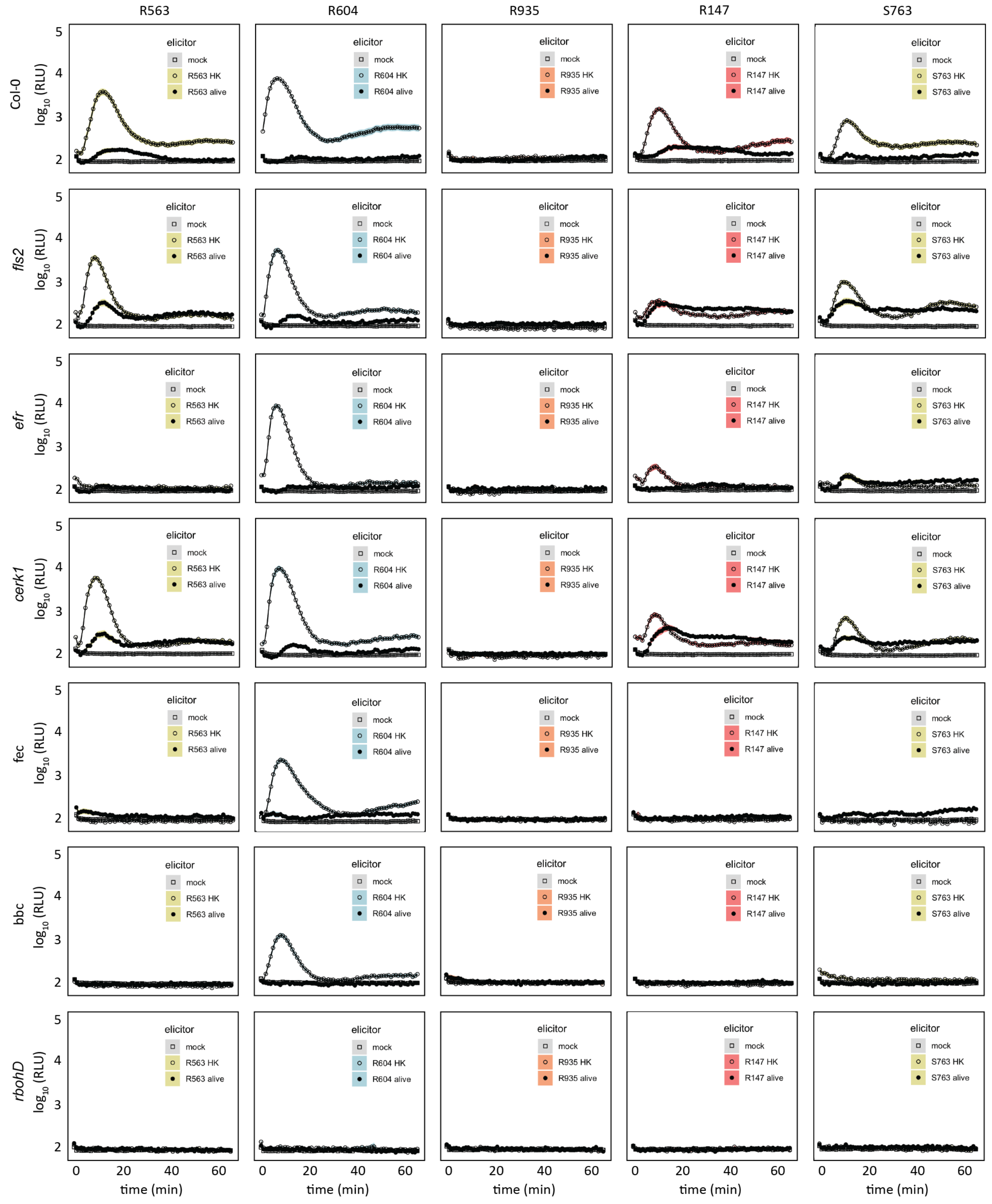

Supplementary Figure S2. ROS burst profile of immune-compromised mutants and Col-0 wild-type plants with commensal bacteria. Leaf discs from 5-to-6-week-old plants were inoculated with live or heat-killed microbiota strains (OD_{600}=0.5) in mono-associations for ROS burst assays. Data from at least 2 independent experiments each with 8 biological replicates were used (n=16). Results are shown as line graphs using Locally Estimated Scatter Plot Smoothing (LOESS) with error bars and shadows indicating the standard errors of the mean.

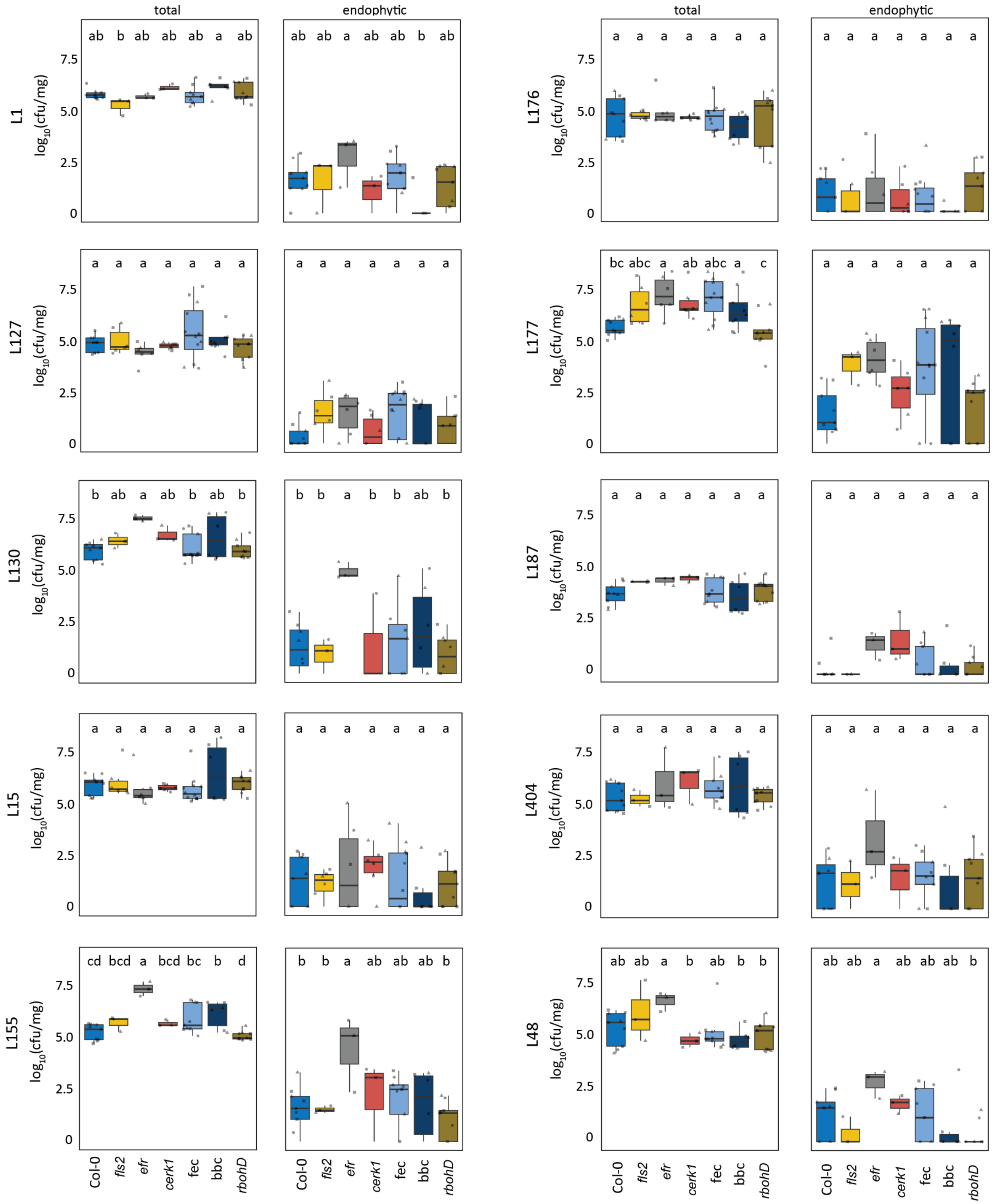
total
endophytic
total
endophytic
L1
L176
L127
L177
L130
L187
L15
L404
L155
L48
log10(cfu/mg)
7.5
5.0
2.5
0
ab b ab ab ab a ab
ab ab a ab ab b ab
a a a a a a a
a a a a a a a
a a a a a a a
a a a a a a a
bc abc a ab abc a c
a a a a a a a
b ab a ab b ab b
b b a b b ab b
a a a a a a a
a a a a a a a
a a a a a a a
a a a a a a a
a a a a a a a
a a a a a a a
cd bcd a bcd bc b d
b b a ab ab ab b
ab ab a b ab b b
ab ab a ab ab ab b
Col-0
fls2
efr
cerk1
fec
bbc
rbohD

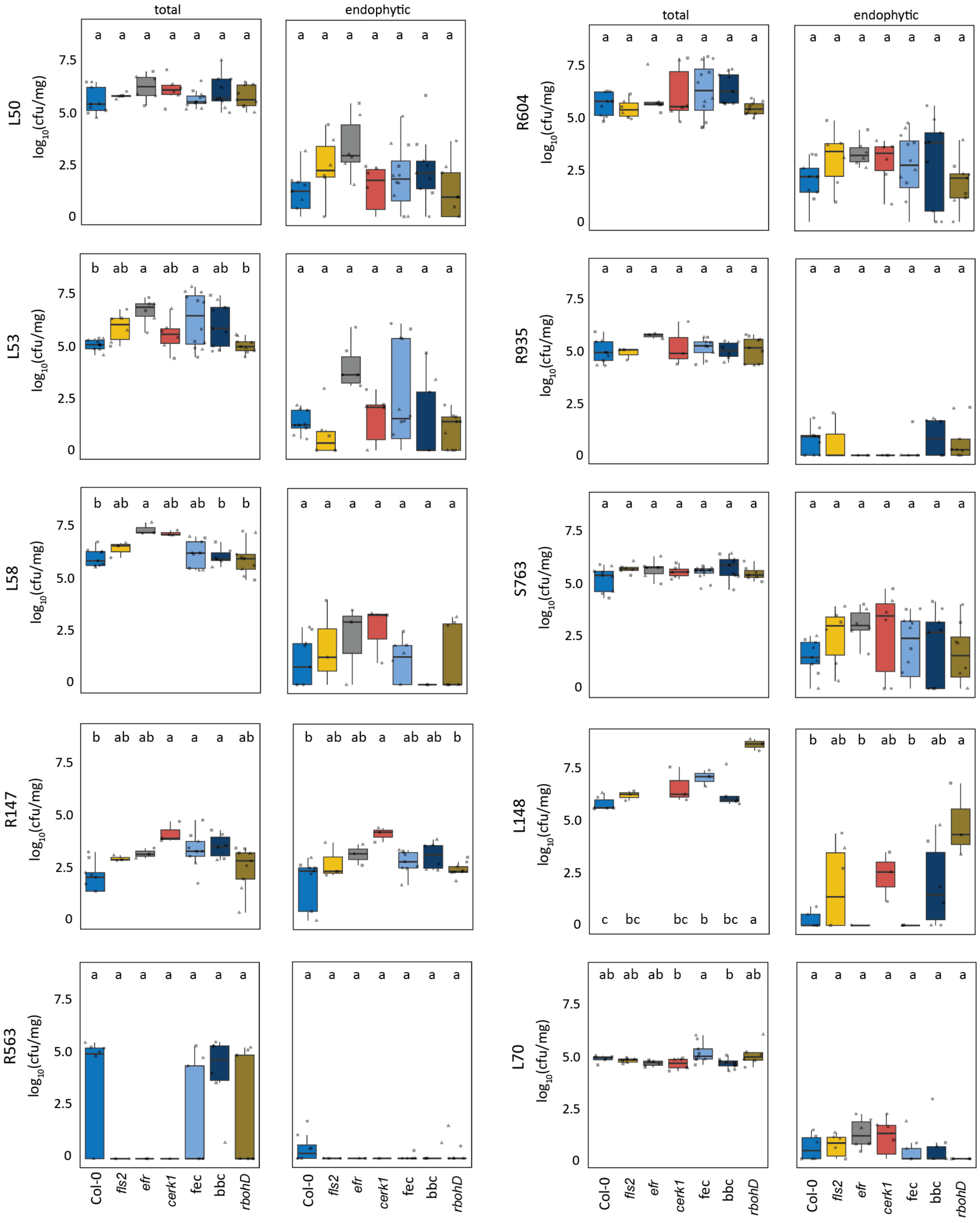

Supplementary Figure S3. Leaf colonization capacities of commensal bacteria on immune-compromised mutants and Col-0 wildtype plants. Two-week-old axenic plants were flood-inoculated with microbiota strains (OD_{600}=0.005) and were plated for colony counts for the total and endophytic leaf compartments at 5 dpi. Data from at least 2 independent experiments each with 3 biological replicates were used (n=9 for Col-0, *fec*, and *rbohD* while 6 for the rest of the mutants). Different letters indicate statistically significant differences (two-sided ANOVA with *post hoc* Tukey's test, $P \leq 0.05$). Results are depicted as box plots with the boxes spanning the interquartile range (IQR, 25th to 75th percentiles), the mid-line indicates the median, and the whiskers cover the minimum and maximum values not extending beyond 1.5x of the IQR.

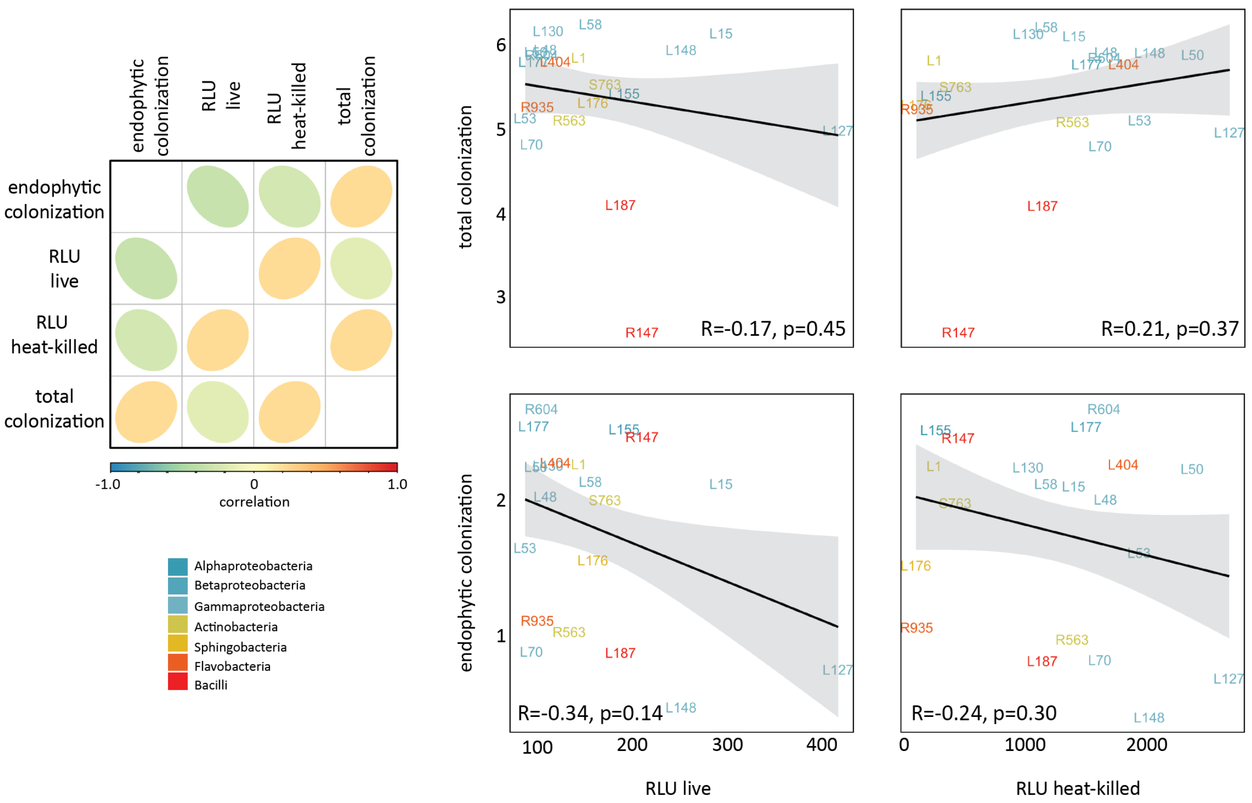

Supplementary Figure S4. The ROS outburst profile and colonization capacities in Col-0 wild-type plants with the microbiota members have poor correlation. Correlational analysis of the capacity of the strain (live or heat-killed versions) to induce ROS and their corresponding colonization profiles in wild-type Col-0 plants (R, coefficient of determination, $p \leq 0.02$). For ROS outburst assay, leaf discs from 5-to-6-week-old plants were triggered with live or heat-killed microbiota strains (OD_{600}=0.5) in mono-associations. For colonization assays, two-week-old axenic plants were flood-inoculated with microbiota strains (OD_{600}=0.005) and were plated for colony counts for the total and endophytic leaf compartments at 5 dpi. Results for correlation were depicted as line graphs using Locally Estimated Scatter Plot Smoothing (LOESS) with error bars and shadows indicating the standard errors of the mean.

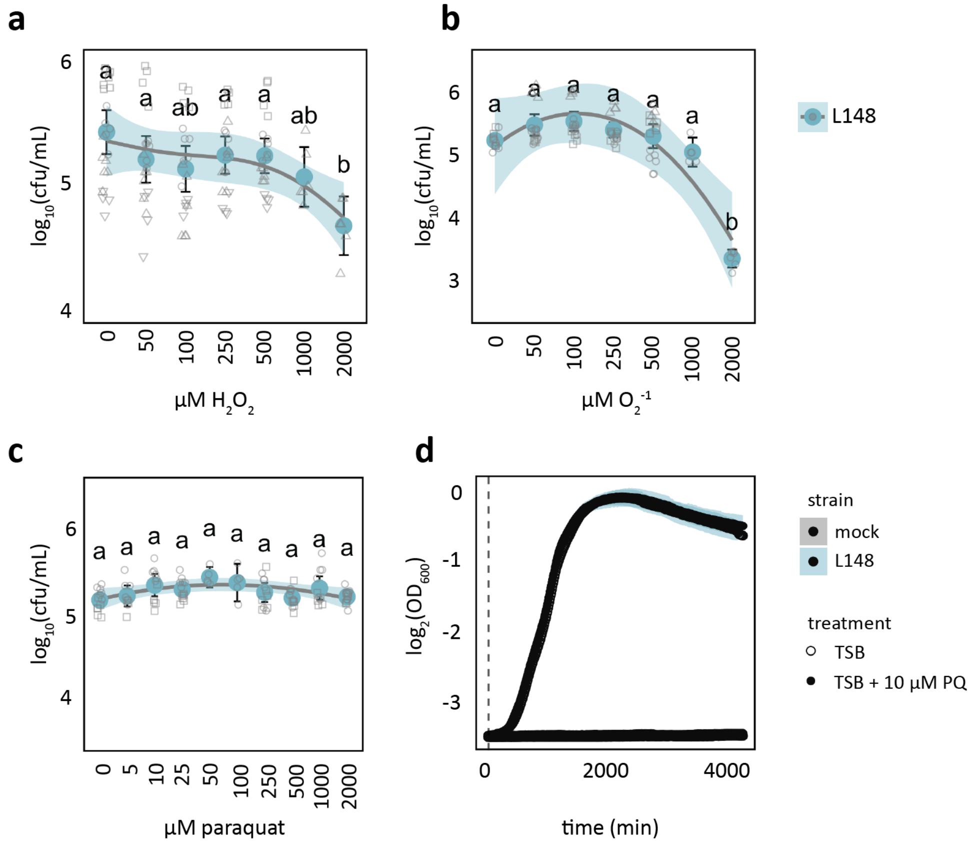

Supplementary Figure S5. *Xanthomonas* L148 is not sensitive to *in vitro* exposure to ROS compounds. **a–c,** Recovery of *Xanthomonas* L148 bacterial cells (initial inoculum OD_{600} = 0.02) upon acute exposure with ROS compounds H_2O_2 (**a**), O_2^- (**b**), and PQ (**c**) in different concentrations (0–2000 µM). H_2O_2 was applied at different doses for 30 min. For O_2^- treatment, 1 mol of xanthine is converted to 1 mol O_2^{-1} with 1 U xanthine oxidase at pH 7.5 at 25 °C in 1 min; reactions were commenced and bacterial cells were sampled at different time points: 0, 2, 4, 10, 20, 60, and 80 min to produce 0, 50, 100, 250, 500, 1000, and 2000 µM O_2^-, respectively. **d,** Growth curves of *Xanthomonas* L148 in TSB upon chronic exposure of 0 or 10 µM PQ for 4000 min. **a-d**, Data were from at least 2 independent experiments each with 3–4 biological replicates (for H_2O_2, n=24, 24, 20, 20, 20, 6, 6 from left to right; for O_2^- , n=16, 16, 16, 16, 16, 6, 6; for acute PQ, n=12; for chronic PQ, n=9). Different letters indicate statistically significant differences (two-sided ANOVA with *post hoc* Tukey's test, $P \leq 0.05$). Results are shown as line graphs using Locally Estimated Scatter Plot Smoothing (LOESS) with error bars and shadows indicating the standard errors of the mean.

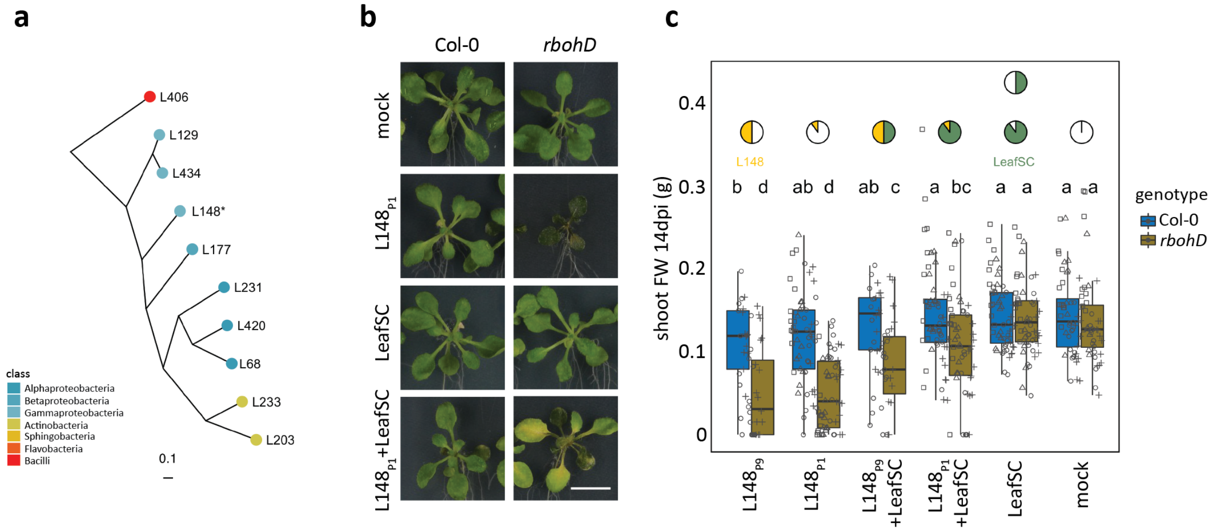

Supplementary Figure S6. *Xanthomonas* L148 pathogenic potential was partially suppressed by the presence of other leaf commensals. a, Phylogenetic relationship of the strains comprising the leaf-derived synthetic community (LeafSC) which consists of strains that are robust and prevalent leaf colonizers, and taxonomically represents diverse members of the leaf microbiota. **b, c,** Representative image (**b**) and the measured shoot fresh weights (**c**) of Col-0 and *rbohD* plants flood-inoculated with mock, LeafSC, $L148_{P1}$ + LeafSC (equal portions of *Xanthomonas* L148 with each strain: L148/LeafSC, 1:9, final OD_{600}=0.01), $L148_{P9}$ + LeafSC (portion of *Xanthomonas* L148 equals the bacterial load of the all strains: L148/LeafSC, 9:9, final OD_{600}=0.01), and the equivalent doses of *Xanthomonas* L148 ($L148_{P1}$ and $L148_{P9}$, P9 is 9 times the dose of P1). The pies indicate the relative proportion of the *Xanthomonas* L148 = yellow and LeafSC = green. White horizontal bar = 1 cm. Data from 2 independent experiments each with 3–4 replicates were used (n=21, 30, 57, 54, 25, 29, 56, 54, 55, 53, 48, and 44, from left to right). Different letters indicate statistically significant differences (two-sided ANOVA with *post hoc* Tukey's test, $P \leq 0.05$).

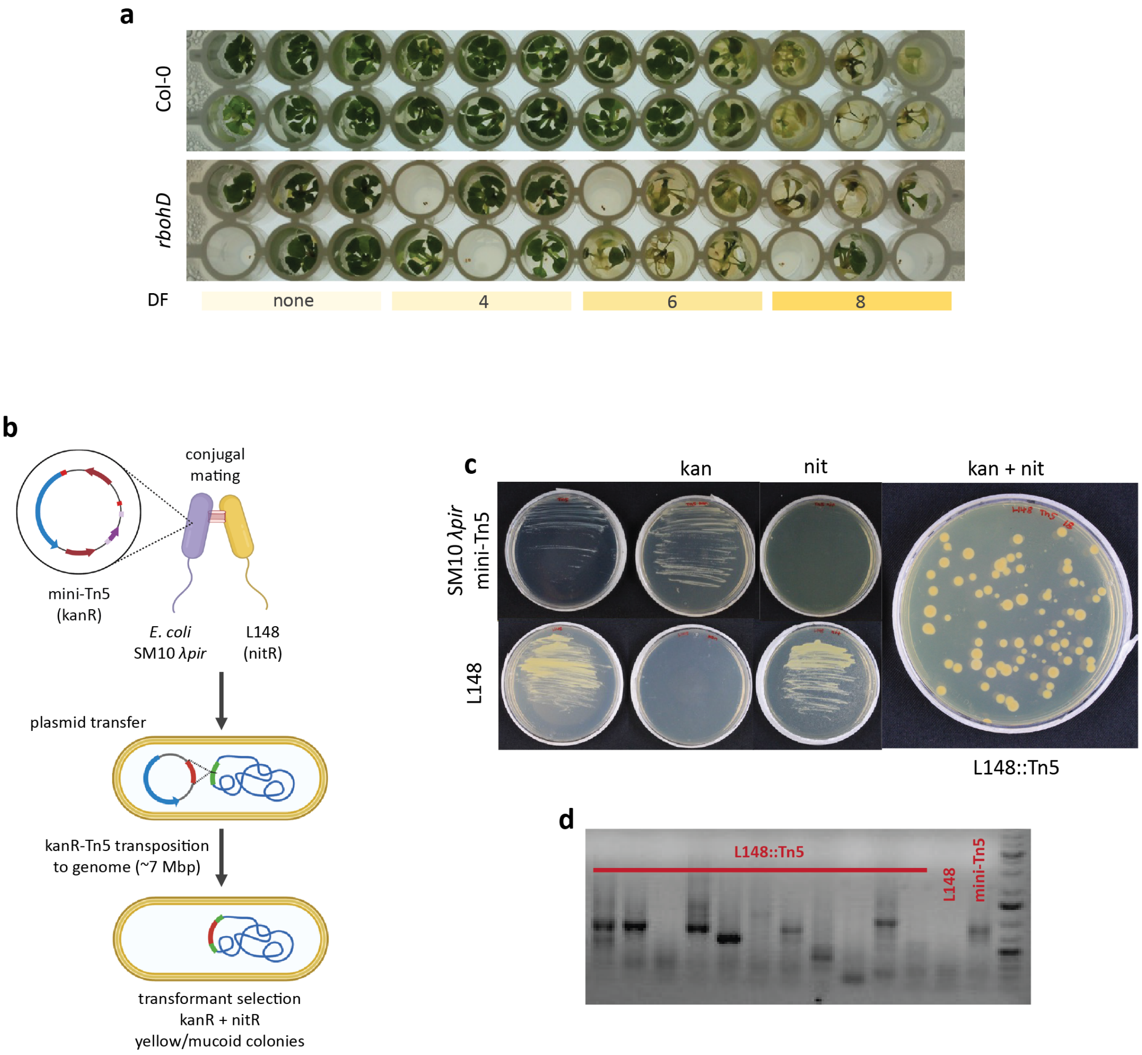

Supplementary Figure S7. Optimization of high-throughput genome-wide screening and generation of the *Xanthomonas* L148::Tn5 mutant library. a, Representative image of Col-0 wild-type and *rbohD* mutant plants inoculated with serially diluted *Xanthomonas* L148 suspensions in the high-throughput 96-well plate format. A dilution factor (DF) of 6 was chosen for the best contrast between Col-0 and *rbohD*. **b,** Schematic diagram of the construction of the *Xanthomonas* L148::Tn5 mutant library via conjugation with *E. coli* harboring the mini-Tn5 plasmid. **c,** Antibiotic resistance of *Xanthomonas* L148, *E. coli* SM10λpir and the *Xanthomonas* L148::Tn5 mutants. The parental strain *Xanthomonas* L148 is resistant to nitrofurantoin (nit, 50 µg/mL in TSB medium) which was used for counter-selection for the plasmid carrier *E. coli.* The mini-Tn5 carrying *E. coli* is resistant to kanamycin (kan, 50 µg/mL in TSB medium) and was used for selecting against the wild-type *Xanthomonas* L148. *Xanthomonas* L148::Tn5 transformants are resistant to both nit and kan in TSB medium. **d**, Electrophoretogram of the genomic transposon insertion PCR validation for the randomly selected *Xanthomonas* L148::Tn5 mutant strains. PCR products were Sanger-sequenced to determine the transposon insertion site. Some of the illustrations were created using BioRender.

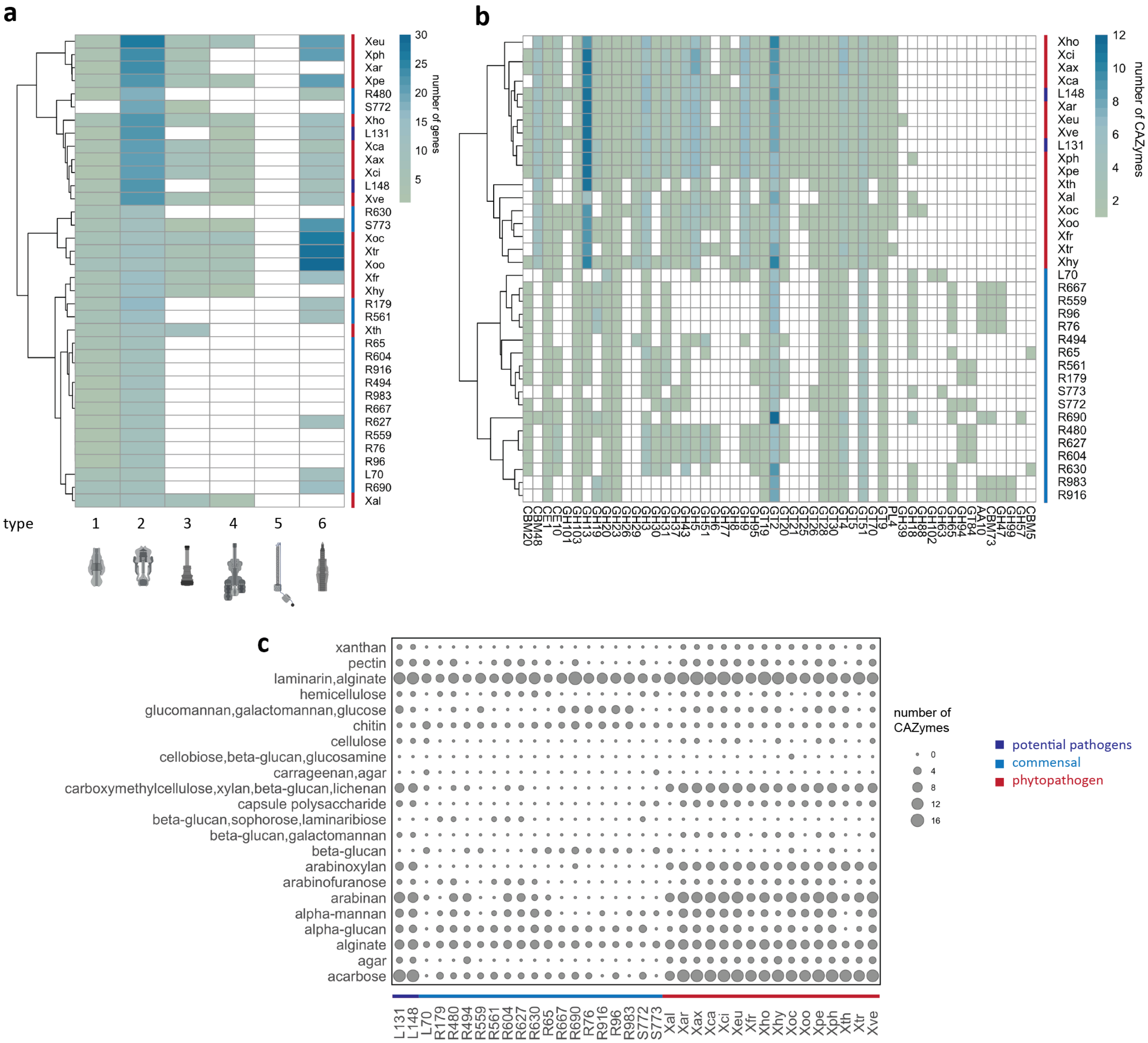

Supplementary Figure S8. Secretion systems and CAZyme repertoire of Xanthomonadales clade. **a-b,** genomic examination of Xanthomonadales members of *A. thaliana* microbiota (20) and pathogenic *Xanthomonas* strains (17): Xal, = *X. albineans*; Xar = *X. arboricola;* Xax = *X. axonopodis*; Xca = *X. campestris;* Xci = *X. citri;* Xeu = *X. euvesicatoria;* Xfr = *X. fragariae;* Xho = *X. hortorum;* Xhy = *X. hyacinthi;* Xoc = *X. oryzae* pv. *oryzicola*; Xoo = *X. oryzae* pv. *oryzae;* Xpe = *X. perforans;* Xph = *X. phaseoli;* Xth = *X. theicola;* Xtr = *X. translucens;* Xve = *X. vesicatoria* ; L148 (in this study) and L131 (Pfeilmeier et al, 2021) are potentially pathogenic. **a,** occurrence of type 1 to 6 secretion systems. **b,** CAZyme repertoire of the Xanthomonadales. **c,** potential substrates of the genome encoded CAZymes. Some illustrations created in BioRender.

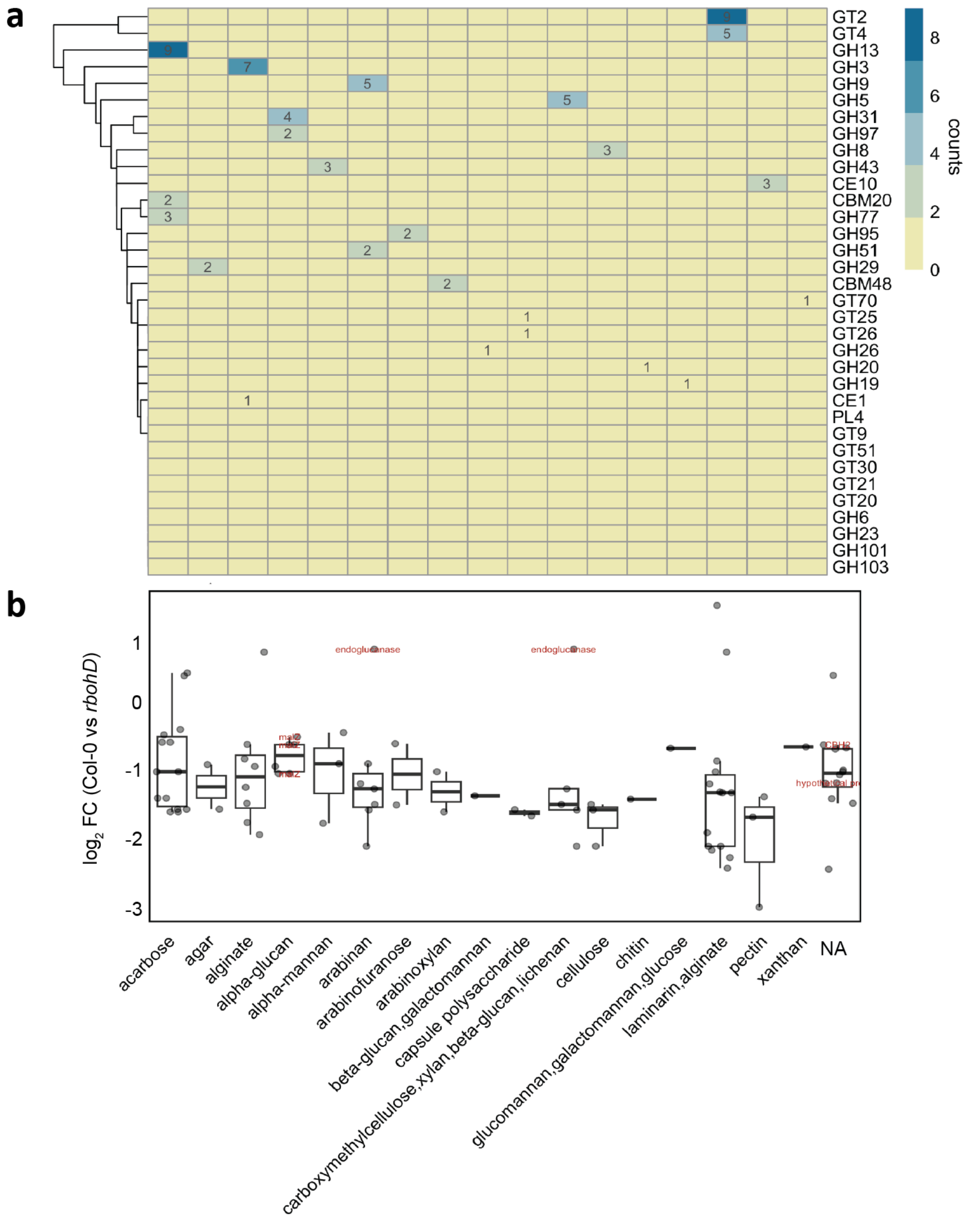

Supplementary Figure S9. Significantly down-regulated CAZymes in Col-0 can potentially degrade plant host cell wall components. **a,** Heatmap representation of the number of significantly differentially expressed CAZyme genes in *Xanthomonas* L148 (Col-0 vs. *rbohD*) with the respective potential substrates. **b**, Log_2 fold changes of the CAZyme gene expression (Col-0 vs. *rbohD*) with their respective potential substrates; *Xanthomonas* L148::Tn5 candidate genes with CAZyme annotations are labelled. Results in **b** are depicted as box plots with the boxes spanning the interquartile range (IQR, 25th to 75th percentiles), the mid-line indicates the median, and the whiskers cover the minimum and maximum values not extending beyond 1.5x of the IQR.

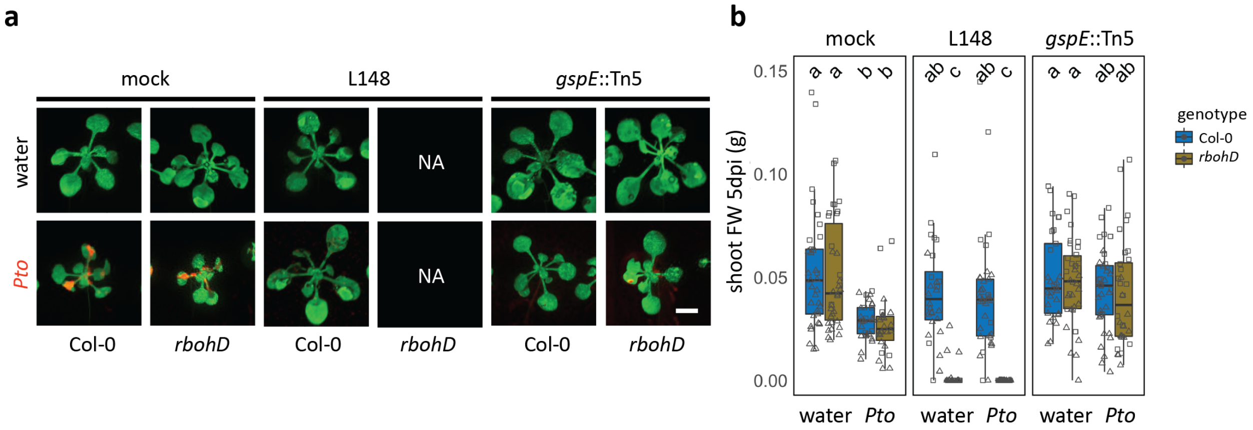

Supplementary Figure S10. Performance of plants pre-colonized with commensals after pathogen invasion. **a**,**b**, Representative images (**a**) and quantification of shoot fresh weight as a plant health parameter (**b**). 14-day-old Col-0 and *rbohD* plants grown on agar plates were flood-inoculated with wildtype *Xanthomonas* L148 and *gspE*::Tn5 (OD_{600}=0.005) for 5 days followed by spray infection with *Pto*. Samples were taken at 5 dpi (2 independent experiments each with 3–5 biological replicates; n=40, 40, 40, 30, 45, 35, 45, 35, 40, 45, 45, 50, from left to right). Red patches in the images indicates colonization by the pathogen. Different letters indicate statistically significant differences (two-sided ANOVA with *post hoc* Tukey's test, $P \leq 0.05$). Results in **b** are depicted as box plots with the boxes spanning the interquartile range (IQR, 25th to 75th percentiles), the mid-line indicates the median, and the whiskers cover the minimum and maximum values not extending beyond 1.5x of the IQR.
