# Supplementary material for: Commensal lifestyle regulated by a negative feedback loop between Arabidopsis ROS and the bacterial T2SS
Source: Nat Commun. 2024 Jan 11;15:456. doi: 10.1038/s41467-024-44724-2 (PMC10784570; doi:10.1038/s41467-024-44724-2)
Supplement: Supplementary file 3 — Description of Additional Supplementary Files [file 41467_2024_44724_MOESM3_ESM.pdf]

Description of Additional Supplementary Files

Supplementary Data 1. List of bacterial strains used and generated in this study.

Supplementary Data 2. List of *Xanthomonas* L148::Tn5 mutant candidates with loss-of-mortality in *rbohD* phenotypes using the high-throughput screening.

Supplementary Data 3. Top table of the DEGs for *in planta Xanthomonas* L148 transcriptome Col-0 vs. *rbohD* colonized plants.

Supplementary Data 4. Clustering membership of the DEGs and the GO term enrichment analysis for the gene clusters.

Supplementary Data 5. List of *Arabidopsis thaliana* wild-type and mutants used in this study.

Supplementary Data 6. List of primers and PCR profiles used in this study.
